# Culture and community: observation of mealtime enactment in early childhood education and care settings

**DOI:** 10.1186/s12966-019-0838-x

**Published:** 2019-08-22

**Authors:** Suzanne Harte, Maryanne Theobald, Stewart G. Trost

**Affiliations:** 1 Institute of Health and Biomedical Innovation at Queensland Centre for Children’s Health Research, Queensland University of Technology, Brisbane, Australia; 2 School of Early Childhood and Inclusive Education, Queensland University of Technology Faculty of Education, Brisbane, Australia

**Keywords:** Eating behaviour, Nutrition, Preschool, Educator, Rituals, Learning, Food preference, Socialisation, Child agency

## Abstract

**Background:**

Establishing healthy eating behaviours in early life has implications for health over the life course. As the majority of Australian children aged five and under regularly attend early childhood education and care (ECEC) services, mealtimes at ECEC settings present opportunities to promote healthy eating behaviors. The purpose of this study was to explore children’s eating behaviours and interactions between peers and educators during mealtimes in ECEC settings, with the aim of constructing a grounded theory of children’s mealtimes in ECEC.

**Methods:**

In-depth qualitative case studies were undertaken at two ECEC centres. Each centre had been assessed as meeting national quality standards and were located in a lower socioeconomic status area. Data collection consisted of direct observation, video recording, written memos, and daily field notes. The analysis included open coding of video recorded mealtimes and field notes resulting in the allocation of initial codes and focused codes. Codes were grouped to form thematic categories and emergent themes. Theoretical sampling was used to identify mealtime interactions exemplifying thematic categories.

**Results:**

Data from 47 mealtimes was available. A grounded theory of children’s mealtimes was developed to demonstrate children’s outcomes at mealtimes. Outcomes were represented by five thematic categories: rituals, learning moments, food preference development, socialisation and child agency. Mealtimes offered opportunities for children to construct a community of peers with their educators by sharing information, stories and occasionally their food. Each centre established its own unique culture within mealtimes observed as the children were involved in routines and rituals.

**Conclusions:**

Mealtimes in ECEC settings are a unique cultural phenomenon co-constructed by the ECEC community of children and educators. The findings highlight the importance of mealtimes as a time for learning and socialization. The routine and rituals of mealtimes provide an opportunity for educators to support the development of healthy food preferences.

## Background

Children’s early experiences with food and mealtimes are important determinants of eating behaviour over the life course. Healthy eating during the early years is essential for optimal growth and development, cognitive functioning and prevention of nutrition related chronic diseases such as obesity, diabetes, cardiovascular disease, and certain cancers [1–3]. Yet, despite the importance of nutrition in the early years, the majority of Australian children aged 2 to 5 years fail to meet guidelines for vegetable intake or consumption of discretionary foods [4].

In countries belonging to the Organization for Economic Co-operation and Development (OECD), the majority of children aged 5 years and under attend some form of early childhood education and care (ECEC). Attendance statistics for ECEC services in Australia show that 71% of 2–3 year olds and 83% of 4–5 year olds attend for an average of 15 h per week [5]. Funding for ECEC services is provided from National and State Government budgets and from fees paid by families [6, 7]. The ECEC setting in Australia is regulated, and ECEC centres are accredited in a process that includes the assessment of seven quality standards encompassing children’s health and wellbeing and the curriculum underpinned by the *Early Years Learning Framework* [8–10]. Thus, ECEC settings are ideally placed to foster a supportive environment for the development of healthy eating behaviors in young children.

Within ECEC settings, educators have a primary care role to provide for the nutritional needs of children in their care. Educators cultivate healthy food preferences through the use of supportive feeding practices and communicating healthy nutrition messages [11–13]. In addition, the eating behaviours of the other children present at mealtimes can influence the type and amount of food consumed [14, 15]. In Australia, all early years training courses provide a module relating to the promotion and provision of healthy food and drinks [16, 17]. Additionally, national guidelines written for ECEC educators suggest that at mealtimes, the role of educator encompasses being a positive role model for healthy eating [18]. Therefore, in the ECEC setting, who is present and how they interact during mealtimes has important implications for the development of healthy eating behaviours. However, little is known about how mealtimes in ECEC settings are enacted, how children and educators interact, or if mealtimes in ECEC settings are aligned with guidelines for healthy eating.

To date, only a handful of studies have focused on children’s mealtimes in ECEC settings. A study conducted in the United States of America (USA) of teacher-child interactions observed that few interactions occurred during mealtimes, with the majority of interactions being directive in nature, including simple statements or commands such as ‘eat your food up’ [19]. Mealtime observation studies undertaken in New Zealand concluded that mealtimes functioned as peer communities that served to socialise children into ECEC culture, reinforce rules and social norms, and create a sense of togetherness [20–22]. A study of children’s responses to educators’ interactions during mealtimes revealed children used their agency to subvert educators’ strategies to shape their food consumption [23]. While these studies reveal different aspects of children’s mealtimes that are important, they were not specifically designed to explore how the actors and their interactions impact on the enactment of mealtimes and children’s eating behaviours. By gaining a better understanding of the functions and outcomes of mealtimes to inform tailored programs for young children incorporating healthy eating interventions, it will be possible to influence children to make choices to positively impact their future health and wellbeing. Therefore, the purpose of this study was to explore children’s eating behaviours and interactions between peers and educators during mealtimes in ECEC settings, with the aim of using the findings to construct a grounded theory of children’s mealtimes in ECEC.

## Methods

The study used an in-depth case study methodology guided by a Constructivist Grounded Theory (CGT) approach to data collection and analysis [24]. The tenets of Ethnography and Symbolic Interactionism are fundamental to CGT methods [24]. As the researcher became embedded within the research setting her role as an ethnographer was established. Children and educators were introduced to her, and she was accepted as a participant during mealtimes. For the researcher, a ‘naturalistic’ style of observation of mealtimes in ECEC was therefore possible. The careful observation, data collection and analysis of the concrete symbols relating to food in this study have been detailed. The way the researcher undertook data collection and analysis simultaneously to understand the interactions between children and their educators while consuming their food during mealtimes shows how symbolic interactionism was applied.

### Cases

Two ECEC centres differing in size and food provision practices participated in the study. The centres were privately owned, recruited from a list of 14 ECEC services identified as meeting national quality standards and serving families from low SES communities in Brisbane Australia. Centre One operated as part of a chain of ECEC centres providing long day care services from 6.30 am to 6.30 pm, Monday to Friday. Food was provided for children’s meals and snacks, and the centre had capacity for 75 children. Centre Two was a solely-owned long day care centre open from 7 am until 6 pm, Monday to Friday. The centre required children to bring their own food in lunch boxes and had capacity for 15 children. Recruitment of children and educators began after written consent was obtained from each Centre Director. Informed written consent was subsequently provided by each educator, and the parents/caregivers of children observed during mealtimes. In keeping with the universal rights of children, child assent was obtained by monitoring children’s willingness to participate in data collection during mealtimes and informing them of features of the study [25]. Table 1 shows the characteristics of the two participating ECEC centres.

**Table 1:** Centre characteristics

	Centre One	Centre Two
Child participants	27 (14 male, 13 female)	20 (12 male, 8 female)
Age of children (years)	4–5	3–5
Educator particpants	5 (1 male, 4 female)	5 (5 female)
Educators’ Early Childhood Qualifications	1 Bachelor degree, 1 Diploma, 1 Certificate, 2 not provided	3 Diploma, 2 Certificate
Centre Ownership	Corporate	Independent
Food provision	Foodservice	Food from home

### Data collection

Empirical data collection methods consisted of direct observation, video recording and continuous written field notes focusing on naturalistic mealtimes. The mealtimes were randomly selected and consisted of breakfast (*n* = 6) morning snack (*n* = 16), lunchtime (*n* = 19) and afternoon snack (*n* = 6). One researcher, an accredited dietitian, was present in the services over a 3 week period. Having the same researcher present in both centres to collect all of the data enabled her to be familiar with the daily activities of each service and how eating occasions occurred within the context of other activities. The resultant data consisted of 24 h and 36 min of video data along with handwritten field notes and memos collected daily over 15 days. Field notes included details of what was seen, what participants were doing, what was said and details of the structure of mealtimes.

Whilst not excluding any children from the usual eating occasion, those without consent (*n* = 3) to participate were not video recorded by adjusting the camera frame to exclude non-participants. Participants were observed during group mealtimes, whether seated at a table, on the ground, or in other arrangements, in accordance with daily routines and rituals. No changes to the natural eating environment were made. The researcher participated in the eating occasion as a helper for educators if required, but refrained from initiating interactions with study participants. While efforts were made to keep the researcher’s influence in the setting minimal, being a participant and observer had the potential to contribute to the constructed reality for all present and may have had the effect of changing some aspects of the mealtimes.

### Data analysis

**Fig. 1:**
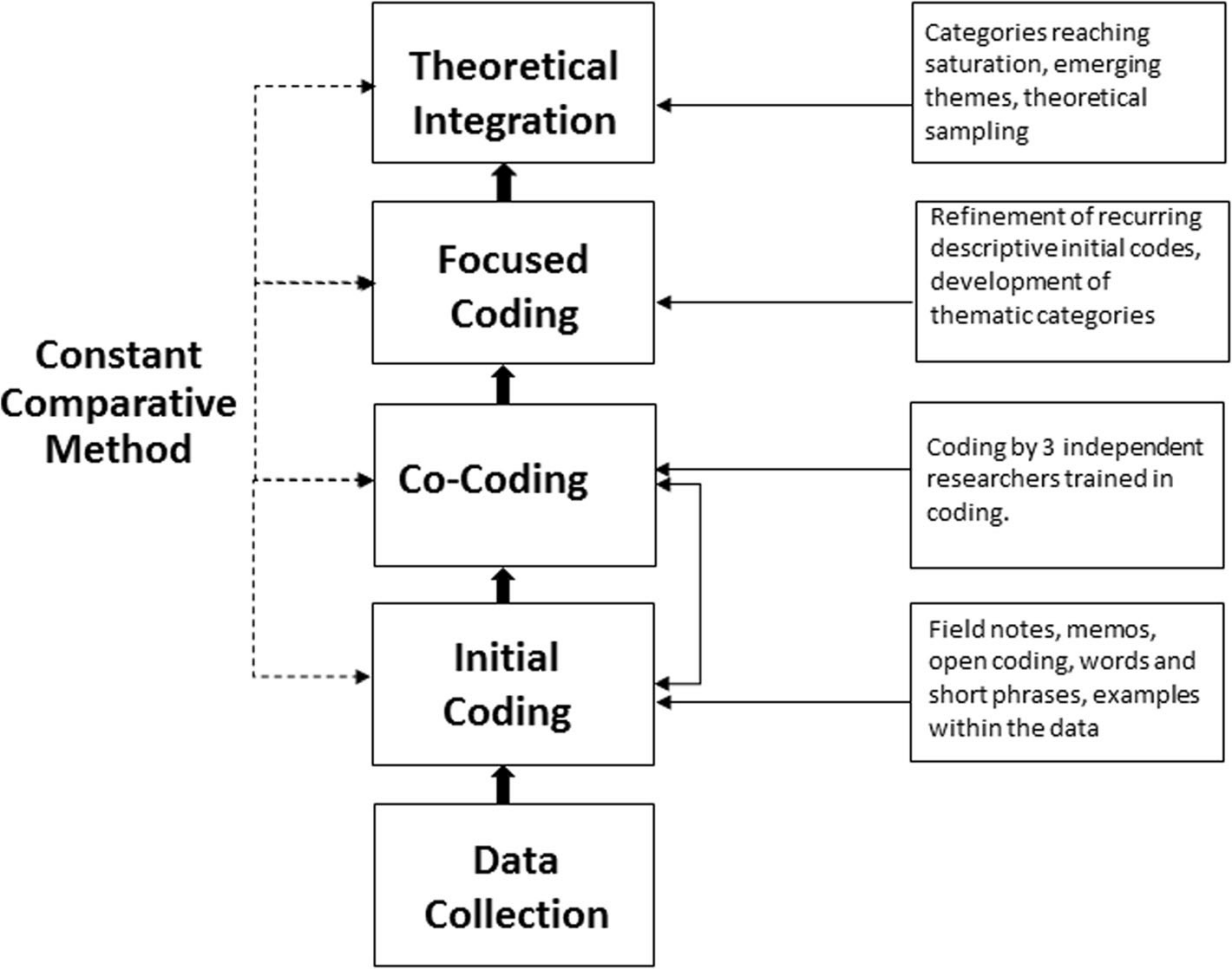
Visual representation of the coding process. Overview of the coding process undertaken in the current study

The process of data collection and coding of data is shown in Fig. 1 below. For a detailed discussion of coding in CGT, the reader can consult Charmaz [24]. Coding started with the principal researcher making initial ‘open’ codes based on observations of all live and video recorded mealtimes and field notes. Initial open codes consisted of words and short phrases describing observations of mealtimes. Mealtime processes and how or why they were adapted on different days were detailed in the field notes, recorded with open codes. The field notes formed the basis of early memos, written with the purpose of describing and developing the analysis as the steps of coding progressed.

The initial codes were further developed and refined through team meetings and discussions [24]. The primary researcher and three researchers trained in open coding simultaneously coded (*n* = 3) randomly selected 8–10 min video segments. The researchers were asked to write down single words or short phrases to code what they observed during the mealtimes in relation to children’s mealtime interactions with each other and with their educators. The co-coded initial codes were then used for comparison with the initial codes developed during data collection. Codes were then discussed as a group with a view to reaching consensus and consistency for how codes were understood. The discussion was video-recorded and notes were then collected from the researchers. If disagreement occurred, the coding was discussed and consensus was made.

Initial coding was followed by focused coding. The researcher used the process of focused coding to synthesise and explain the data (e.g., video data, field notes, and memos) using descriptive and/or frequently recurring initial codes. As focused coding was refined, thematic categories were developed and grouped. Saturation of thematic categories occurred as focused codes were observed to recur, leading to the emergence of themes, allowing for the final step of theoretical integration to be undertaken [24]. The emergent focused codes were associated with behaviours and actions of the children and the educators, including language, conversation topics, seating positions, and physical setup. Theoretical sampling was undertaken to refine coding of thematic categories, and in constructivist grounded theory refers to the process of seeking and collecting pertinent data to elaborate and refine categories in an emerging theory [24]. Theoretical sampling, using examples from the video data, field notes and descriptive memos, demonstrated saturation of the thematic categories. The analysis process culminated in the construction of a grounded theory of children’s mealtimes described in the results below. Table 2 shows the detailed coding scheme developed for the analysis of observed mealtimes.

**Table 2:** Results of data analysis and CGT coding technique

Emergent Theme	Thematic Categories	Focused Codes	Initial Codes
Culture and community of mealtimes	Rituals	Rules, routines, flexibility, transition, local practices, food environment: physical, emotional atmosphere, positional power imbalance, surveillance, control, tension.	Routine, transition, temporal influence (hurry), sleep/rest association with routine: post-prandial expectation, environmental controls: table configuration, inside/outside, placement, lighting, music, loud noise, utensils: absence and adult-size and age appropriate, cleaning, packing up, supervision, monitoring, emotional tone: tension vs relaxed, social, conversation, compassion, subversion, individual attention.
	Food preference development	Food availability, guided food choice, control, information sharing, perception of healthy eating, discretionary food, utensil competency,	Menu, food service, supervision, monitoring, assistance with containers, controlling feeding practices, supportive feeding practices, healthy food, unhealthy food, food sharing, subversion, culturally diverse food, food safety, waste food, sustainability,
	Socialisation	Role model, supervision, monitoring, compliance, socialisation, educator influence, control, social interaction, physical assistance, competency with utensils, learning opportunities (and missed..), extended learning, home culture, judgement, moral work, community of educators, community of children, peer influence.	Peer modelling, peer-negotiation, dispute, behavioural expectation, social mealtimes, moral work, extension to home (informing – good/bad behaviour), social commentary, behavioural threats, consequences, play at mealtimes, food sharing, imaginative play, coercion, communication, normalized behaviour, behavioural comparison, tension, social, reward for behaviour of eating, atmosphere, decision-making, inclusion, exclusion, separation/isolation.
	Learning moments	child agency, control, socialisation, rules, communication, social eating, food choice, novel food, peer influence, learning opportunities, passive learning, extended learning, intentional learning, food systems, food values, food connections.	Peer influence, storytelling, information sharing, subversion, peer modelling, food sharing, food play and role play, social interaction vs behavioural expectations, play at mealtimes, relaxed vs controlled mealtimes, sustainable and interactive food systems, food cycle, reading books.
	Agency: child agency, educator agency, caregiver agency, tension	Communication, food availability, extended learning, food choice, food selection, sensory engagement, food preference development, educator feeding practices, child eating behaviour valued food, novel food, controlling feeding practices, supportive feeding practices, conformity, regulation – child educator, non-regulation – caregiver, educator influences, peer influences, caregiver influences, learning opportunity.	Foodservice, lunchbox, home food, cultural food, food preparation demonstration, food safety, food likes and dislikes, appetite, hunger and fullness cues, educator verbal cues (finish eating), praise, instruction, portion control, food as reward + (discretionary food as reward), coercion, guided food selection, child chooses to sit/not to sit.

## Results

The analysis identified a single emergent theme as saturation of coding occurred - cuture and community of mealtimes. This emergent theme was constructed from five thematic categories: 1) rituals, 2) learning moments, 3) food preference development, 4) socialisation and 5) child agency. The thematic categories and emergent themes are described below.

### Thematic categories

#### Rituals

Rituals developed in ECEC services help children by establishing routines and signalling transitions to everyday events [21]. Rituals were prominent in the mealtimes observed in this study. Prior to their midday mealtime, children were observed to engage in specific routine activities as part of the transition from planned activities to food being served. In Centre One, routine activities for children included making their beds, washing their hands, taking a seat at the table and being served. Educators were observed giving the children guidance, prompts and instructions, reminding them of what was expected as part of the routine. Once they were served, the children then started eating. In Centre Two, the use of rhyming words to help children to transition through the routine of washing hands as an antecedent to the mealtime ritual is shown in Extract 1.

**Extract 1:**

*Miss Rachel: "Willowby Wallaby Woo, an elephant sat on you, Willowby Wallaby Woo, an elephant sat on … “Martin”*

*Martin leaves the group to go to wash his hands*

A repetitive rhyme spoken by educators and children before eating was an example of a meal time ‘transition’ ritual in Extract 2.

**Extract 2:**

*All: “Shh, Shh, Shh, Ready? Good food, sweet treats, good friends, let’s eat”*

In Centre Two children and at least one educator were seated each day, eating and interacting in a variety of ways. The ritual continued when children placed their lunch boxes on a trolley when they were finished their food. At the completion of the midday meal, children left the eating area, to make a transition to rest time having prepared their beds earlier, concluding the mealtime ritual.

#### Learning moments

Unplanned and planned learning moments were observed at mealtimes, presenting opportunities for learning extension [26, 27]. Learning occurred in a variety of ways, and at mealtimes, concepts of nutrition education and food production were observed and linked to children’s reading books available in the centre. In Centre Two, educators asked questions about the foods children brought from home. Extract 3 is part of a conversation, in which an educator discusses a child’s quiche in terms of the main ingredient (eggs). She describes eggs from a farm as being ‘yummier’ and makes a reference to the children’s book *Green Eggs and Ham* [28].

**Extract 3:**

*Nathan: “What is he eating?”*

*Miss Leanne: “He’s having quiche.”*

*Brady: “I love quiche.”*

*Miss Leanne: “You do love quiche, yeah. It’s based on egg Nathan.”*

*Nathan: “Did you know you get the egg from a chicken, and they poop it out?”*

*Miss Leanne: “They don’t really poop it out, they lay the egg”.*

*Billy: “Ham, ham is pig”*

*Miss Leanne: “Ham is pig, yes, ham is pig, so is bacon, so is pork.”*

*Brady: “Give me all your eggs, and green eggs”*

*Miss Leanne: “Green eggs? Green eggs and ham?”*

*Brady: “Yeah”*

#### Food preference development

Food preferences are an important influence on children’s food choices at mealtimes [29]. Children’s food preferences, their likes and dislikes along with food availability can affect the amount they eat [30]. In Centre One, children’s food preferences were influenced through exposure to the foods served from the kitchen. In Centre Two, the observation of peers’ lunchboxes and discussion about the food children had brought with them provided awareness of a variety of foods. Educators used a range of communication strategies to guide children’s food choices during mealtimes. Strategies included the sharing of information about food children had brought, educators sharing their perceptions of healthy foods and what foods were deemed to be appropriate for mealtimes. At Centre Two, educators introduced children to novel foods and encouraged them to eat them as shown in Extract 4.

**Extract 4:**

*Miss Leanne: “Yes, Goji Berries are yummy”..*

*Brady: “Can I hold one?”*

*Miss Kirsty: Miss Leanne’s super food, here we go."*

*Miss Leanne: Would you like to try a Goji berry? (Brady shakes his head). No thank you, no, ok, nice manners.*

#### Socialisation

Socialisation is the process where children are taught the behavioural expectations of the centre including prosocial behaviour such as the use of positive language and actions [31, 32]. Aspects of daily life and the norms of the ECEC centres were discussed as opportunities arose during mealimes. Children’s observation of peers’ behaviour at mealtimes provided examples of modelling of behavioural expectations and were positively reinforced by educators in front of the other children. Educators were observed supervising and monitoring children’s behaviour and providing assistance to children for utensil competency and opening containers. Verbal interactions were allocated open codes that included moral work, imparting judgement and social commentary. In the extract below, the educator justifies how a child’s foods should be consumed throughout the day by linking ideas of how attending ECEC is aligned with the social norms of working parents.

**Extract 5:**

*Miss Leanne to Violet: “Eat your cheese sticks, save your Cruskits for afternoon tea – because your Mummy and Daddy get here late.”*

*Violet: “Why do my Mummy and Daddy get here late?”*

*Miss Leanne: “Because they work Sweetheart, it’s just what their work does, but I just wanted you to have something a little bit bigger at afternoon tea.”*

Mealtimes were a time of community for children, when they chose who they wanted to sit with and share stories. In Centre Two, children engaged in play at mealtimes, and despite the expectation for having good manners at mealtimes, this included using food for play such as having food fights. Children developed an understanding of the world through the use of imaginative play to engage with peers and role play how families share meals. Extract 6 demonstrates how mealtimes served as opportunities to reinforce behavioural expectations including identifying acceptable behaviour and respect for each other.

**Extract 6:**

*Nathan moves toward Violet and pushes her arm, she whimpers, “Don’t” and Nathan growls and moves his hands like claws toward her, pouncing at her shoulder. Miss Leanne approaches: “Nathan, Nathan, hands to yourself please Buddy”, and sits him down, bodily moving him in his chair further around the table,*

*Violet continues eating.*

*Miss Leanne: “Are you okay Violet?” Violet nods and finshes eating.*

#### Child agency

Child agency refers to having opportunity to make independent decisions in everyday situations [33]. The notion of children being involved in developing a sense of agency to influence their everyday activities while attending ECEC services is encouraged [9]. During mealtimes, children were observed to have opportunities to exert agency. Whilst children had limited input in the preparation of food, they were seen to be able to choose what and how much to eat. In Centre One, mixed sandwiches were served on a platter in the middle of the tables during a lunch mealtime. Children were encouraged to use tongs to self-select their preferred filling and a number of sandwiches according to their appetite. Whilst a foodservice was a feature of the mealtime, children were permitted to bring food from home in a lunch box. Those children bringing a lunch box were able to choose between the menu selection or food from their lunchbox, supporting their agency. At times however, educators provided guidance for children, resulting in a reduction of children’s agency for their food choices. In Centre Two, where food was prepared at home, educators still had influence over what was eaten by commenting on children’s food choices. In Extract 7 below, the educator is giving guidance about food choices at specific times throughout the day. In Extract 8, she also provides information about how a sandwich should be prepared in accordance with the local practices at the centre. Both examples demonstrate how children’s agency was thwarted.

**Extract 7:**

*Daisy: “I want it (sticky date pudding) after my morning tea.”*

*Miss Leanne: “That would be afternoon tea, okay. So you’ll have your yoghurt and strawberries now.”*

**Extract 8:**

*Miss Leanne: “Show me. It’s a Vegemite sandwich. You need to get your crusts left on your sandwiches, tell Daddy don’t forget the crusts.”*

### Emergent theme

The emergent theme identified was culture and community of mealtimes. Culture related to ECEC mealtimes was supported by the many processes of mealtimes, and in particular thematic categories of learning moments and rituals. Additionally, mealtime interactions associated with the socialisation of children underscored the unique culture at each ECEC centre. Observations highlighted how guidance from educators was given to children to reinforce the way mealtime routines were uniquely enacted in each service. The transition activities preceding and proceeding mealtimes were repeated on a daily and familiar to all children who attended. The development of a unique culture in each centre provided children a reliable frame of reference for the enactment of mealtimes at the centre they attended.

Depending on the foodservice menu for Centre One, the cultural features of the foods served were determined by decisions relating to palatability, budget and food safety considerations. The food was cooked, prepared and then served while the children were seated at tables. Food was brought from home at Centre Two, thus children had potential for broader cultural influences in relation to food selection and food availability. For children attending Centre Two, the cultural features of the available foods were determined by the food preferences and decisions of the child and their family, guided by the unique features of home life. Whilst all families were given general guidelines for packing lunchboxes, the types of foods included in lunchboxes varied.

Community of mealtimes emerged as the second part of the theme, in particular because of the people present during mealtimes. Educators had a central role during mealtimes, contributing actively to the ECEC community at mealtimes. Educators were observed eating with the children and providing social interaction, opportunities for learning and guidance to support healthy food preferences. Children had opportunities to participate in their community of peers by sharing information and stories. Children were observed sharing food brought from home when educators were distracted, and giving disliked food away so educators thought the food had been eaten, demonstrating their willingness to work together to solve problems despite the local mealtime expectations.

### Constructed grounded theory

**Fig. 2:**
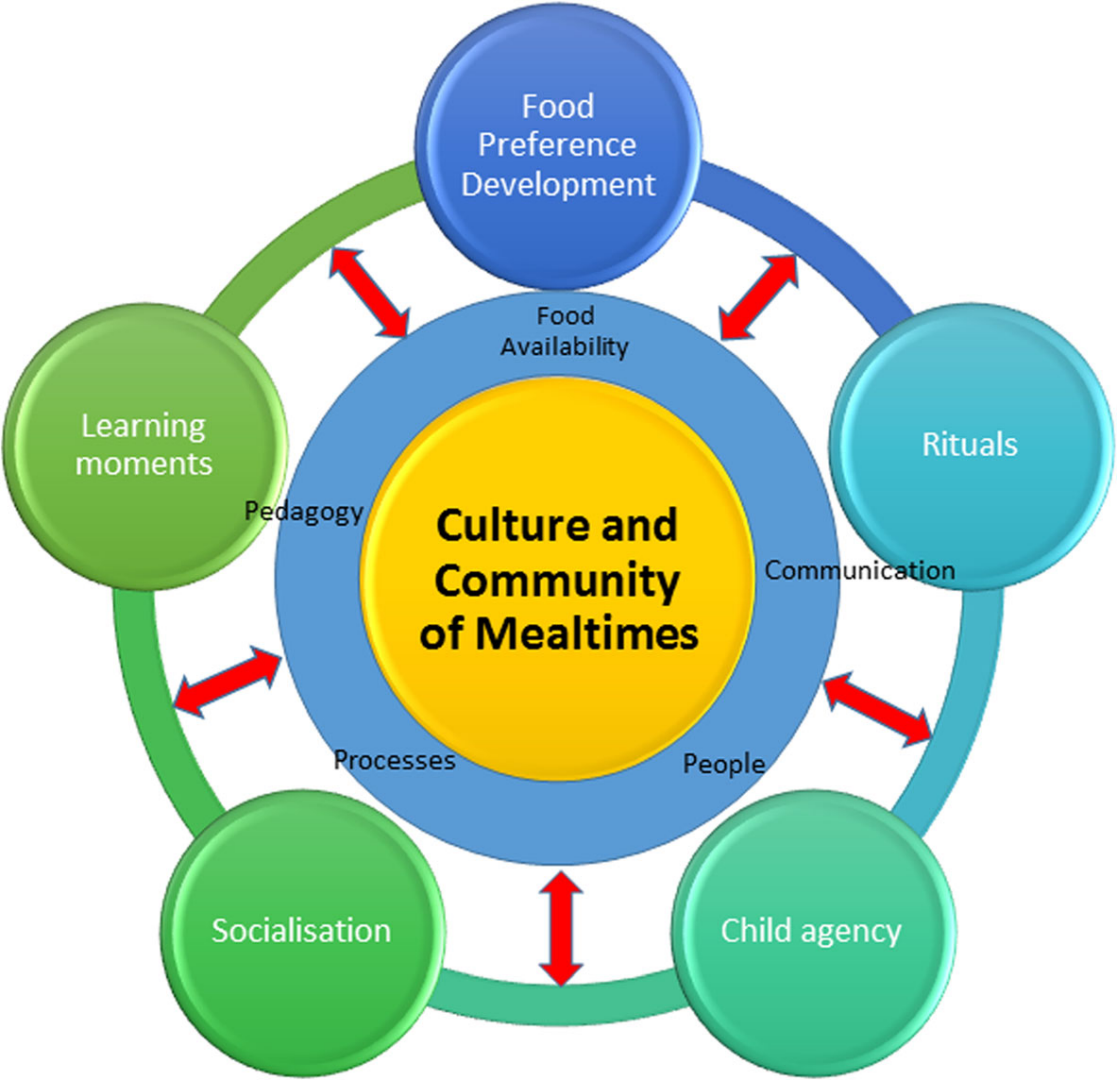
The culture and community of ECEC mealtimes

As a result of the iterative analyses reported above, the following constructed grounded theory of Children’s Mealtimes in ECEC emerged. The five thematic categories formed the outer layer of the model, contributing to the emergent theme of culture and community of mealtimes shown at the centre of the model. The model depicts a micro-system of mealtimes enacted in ECEC settings, highlighting the inter-related components of the grounded theory. The inner and outer layers were linked by processes or entities collectively referred to as “mechanisms of mealtimes”. The mechanisms, identified through focused coding, were present in both services and comprised communication, people, processes, pedagogy and food availability. Communication refers to the verbal and physical interactions between educators and children during mealtimes. The people or actors in the setting are the educators and children participating in the enactment of mealtimes. Processes consist of the activities undertaken to differentiate mealtimes from other ECEC activities such as sitting down at a table, collecting water bottles and lunchboxes, and disposing of uneaten food. Pedagogy refers to educators’ teaching strategies undertaken during mealtimes. Food availability refers to the food served to children and educators and the manner in which it is served - either in a lunch box or through a foodservice (Fig. 2).

## Discussion

Children’s early experiences with food and mealtimes are recognised as central to establishing patterns of lifelong healthy eating behaviours [1, 4]. However, despite mealtimes being a feature of each child’s day whilst attending ECEC services, little is known of how mealtimes are enacted in this setting. Gaining a better understanding of how mealtimes are uniquely enacted within the ECEC setting provides a way for guidelines to be developed so that strategies to support healthy eating can be tailored effectively for different communities. The present study observed children’s eating behaviours and interactions between peers and educators during mealtimes with the aim of constructing a grounded theory of children’s mealtimes in ECEC settings. The resultant theory conceptualized the enactment of mealtimes as a micro-system encompassing outcomes and mechanisms that uniquely contribute to the community and culture of each Centre. Through the analytical processes of focused coding, theoretical sampling, and theory building, a key emergent theme was revealed: culture and community of mealtimes [24]. The identification of the theme highlights the interdependence of people and processes in the ECEC setting, especially during mealtimes. As the theme occurred consistently through the process of analysis, the community of mealtimes, co-constructed by children and their educators, provided opportunities for observational learning, supported child agency, and aided in the development of healthy food preferences. Within each centre, the routines and rituals associated with mealtimes contributed to a unique sense of culture by reinforcing behavioral expectations, teaching children the social norms for commensal eating, and exposing children to a variety of foods. The identification of a major theme therefore presents an opportunity for future exploration of how the people and processes are inter-related within the mealtime micro-system, and the enactment of mealtimes.

The findings have a number of important implications for practice and policy. Opportunities exist to build on educators’ capacity to reinforce healthy eating messages during learning moments that occur at mealtimes by linking evidence-based guidelines to teachable moments that occur every day. A deeper understanding of mealtimes as a micro-system within the ECEC setting can help educators develop healthy eating policies and practices that take into consideration how the rituals and interactions with their peers and educators affect children’s food preferences, social and learning outcomes. Opportunities may include the development of new tools to measure educators’ interactions with children during mealtimes. Educators and children will benefit from healthy eating strategies that take into consideration food preference development and rituals that help mealtimes to be ‘social and relaxed’. Consistent use of supportive feeding practices in ECEC settings has potential to minimise confusion for children about mealtime expectations and to support children’s ability to self-regulate their dietary intake according to their internal hunger and satiety cues. As children develop knowledge of their own hunger and satiety, this in turn will have implications for their food choices and later life health outcomes [34, 35]. From the assessment of mealtime interactions, it may therefore be possible to design and implement professional development programs to help educators use communication strategies which promote effective feeding practices.

Our findings are consistent with those of Holsten et al. [29] who developed a theoretical representation of how children engage in making food choices during mealtimes enacted in the family home. The authors identified role modelling, routine, food preferences and food availability as key factors, and these factors were also observed to be important for mealtimes enacted in ECEC settings. That children and their educators co-constructed a community of mealtimes through sharing a meal, group rituals, and storytelling is also consistent with the findings of Sobal et al. [36, 37] and Giacoman [38] who studied the communal factors of family mealtimes, described as commensality. Our findings therefore support the importance of community and peer culture in the socialisation of children at mealtimes with potential to have influence upon children’s food preferences. The identification of a unique micro-system to represent ECEC mealtimes is intrinsic to the capacity of educators to support healthy eating for children. Our analyses indicate that mealtimes at ECEC therefore serve a greater purpose. Mealtimes are a time when children are eating in a communal activity and this represents an opportunity to promote healthy eating as an expectation for the children. Collectively, the findings highlight the intricacies of what is happening for children in ECEC particularly in relation to the outcomes of socialisation, food preference development and learning.

Socialisation emerged as thematic category in this study. Our study found that children ate together, and normative behavioural expectations were identified and reinforced for the children to adhere to. Behavioural expectations were consistent with the results of a study conducted in New Zealand ECEC’s, which suggested that mealtimes were a time when children demonstrated togetherness and unity [20]. Educators had an important role for managing children’s behavior at mealtimes. Children’s mealtimes were observed in an ethnographic study identifying consistent themes related to how children were engaged by their educators [22]. Strategies related to teasing, humour and tenderness were found to be effective for children to understand and participate in ECEC mealtimes [22]. The findings from this study suggest that socialisation was essential for children to contribute to their ECEC mealtime community of peers and educators. Teachers used strategies designed to enculturate children to mealtime routines by using rhyming words to identify a ritual as part of an established routine. However, at times rules associated with mealtimes were subverted such as was observed when children engaged in food fights. This tendency for subverting rules was also found to be a feature of children’s communal mealtimes in relation to children’s agency in another study [23]. By observing and analysing mealtimes, the importance of mealtimes as an opportunity for teaching children about behavioural expectations and managing antisocial behavior was highlighted. Children demonstrated acceptance of rules and rituals through their participation in the mealtimes, and therefore, participation could determine not only a measure of success for the routine or ritual, but also the mealtime experience.

Our findings lend strong support to the notion that children’s food preferences can be influenced by educators and peers. Having food provided for all children via a menu, or foods provided from home, complying with guidelines aligns with the normative dimension of commensal eating [36, 38]. The expectation that the foods served during the mealtime will be eaten, reinforced by the peer influence of children complying with the norm, has the potential for new foods to be more readily accepted. The development of food preferences has been shown to be influenced by early life cultural experiences in the home [29]. In the current study, the cultural experiences of each of the actors had the potential to enrich the experiences of other children through having opportunities for introduction to new foods. Increasing children’s awareness of novel foods has importance for two reasons. Firstly, in a culturally diverse country like Australia, children being exposed to a variety of foods has implications for developing preferences for a wide range of foods, including traditional foods from countries that may not be available at home and vice versa. Secondly, children may be provided access to a wider variety of healthy foods for the first time. Therefore, enculturation to the ECEC mealtime goes beyond the acceptance of ritualised processes, it also provides support for the acceptance of foods that are generally accepted by the peer group.

The study has a number of strengths. Firstly, it is the first to explain the enactment of mealtimes as a micro-system existing within the context of ECEC. By adopting an established methodological approach, underpinned by iterative and comparative analysis, themes and thematic categories were developed in a way that was generalizable between the two centres, despite differences in the way food was provided. Secondly, adopting a deductive method of analysis allowed the themes to emerge without the influence of an existing theoretical lens, avoiding a tendency to reach expected or predetermined outcomes. Thirdly, the identification of cultural overtones and food provision as a central tenet to the enactment of mealtimes illustrates food as a symbolic artefact [24]. Valuable insights from this analysis have highlighted opportunities for future studies to further examine mealtimes and the symbolic interaction of food provision in ECEC. Finally, the CGT approach acknowledges the researcher’s role in co-constructing the phenomenon being examined to reduce the potential for researcher bias.

Having identified the strengths of this study, a number of limitations should be considered. Firstly, mealtimes are an activity that occurs dynamically and can be influenced by other factors such as the ECEC workforce, seasonal effects and the concurrent personal or social events impacting the children. Secondly, these findings may not be considered generalisable as the study was conducted in two centres in a specific area of Brisbane, Australia and may not be reproducible in a wider range of ECEC settings. Finally, the presence and acceptance of the researcher as an ‘insider’ is not guaranteed. The researcher’s presence in the setting had potential to change the usual enactment of a mealtime. Participants may have wished to portray mealtimes in a favourable light especially as data collection techniques included video-recording.

## Conclusions

Our findings suggest that the enactment of mealtimes can be viewed as a micro-system encompassing mechanisms and outcomes that uniquely contribute to a mealtime culture and community in the ECEC centres. Having identified social interaction as integral to the mealtime micro-system, future research will benefit from an exploration of educator, parent and child perceptions. These perspectives will provide additional insights into the co-construction of a unique culture and community at mealtimes. Future studies of other age groups such as infants, or studies of ECEC’s operating in other jurisdictions with different regulatory frameworks would enable a broader understanding of mealtime enactment.

## Data Availability

The datasets generated and analysed during the current study are not publicly available due to ethics restrictions applicable to privacy in relation to sharing images of children, but are available from the corresponding author on reasonable request.

## Abbreviation

ECEC: Early childhood education and care

## References

[1] Uauy R, Kain J, Mericq V, Rojas J, Corvalán C (2008). Nutrition, child growth, and chronic disease prevention. Ann Med.

[2] Chiuve SE, Fung TT, Rimm EB, Hu FB, McCullough ML, Wang M, Stampfer MJ, Willett WC (2012). Alternative dietary indices both strongly predict risk of chronic disease1-3. J Nutr.

[3] World Health Organisation (2016). Report of the commission on ending childhood obesity.

[4] AIHW (2018). Nutrition across the life stages.

[5] Australian Bureau of Statistics (2015). Childhood education and care, Australia, June 2014.

[6] Australian Government (2015). Child care in Australia - a quick guide.

[7] Australian Children’s Education and Care Quality Authority (2018). Early childhood and child care in summary.

[8] Australian Children’s Education and Care Quality Authority (2017). Guide to the national quality framework.

[9] Department of Education Employment and Workplace, editor. Canberra: Belonging, being & becoming, the early years learning framework for Australia; 2009.

[10] Australian Government (2011). Education and care services national regulations.

[11] Sharma S, Dortch KS, Byrd-Williams C, Truxillio JB, Rahman GA, Bonsu P, Hoelscher D (2013). Nutrition-related knowledge, attitudes, and dietary behaviors among head start teachers in Texas: a cross-sectional study. J Acad Nutr Diet.

[12] Erinosho TO, Hales DP, McWilliams CP, Emunah J, Ward DS (2012). Nutrition policies at child-care centers and impact on role modeling of healthy eating behaviors of caregivers. J Acad Nutr Diet.

[13] Hughes SO, Patrick H, Power TG, Fisher JO, Anderson CB, Nicklas TA (2007). The impact of child care providers’ feeding on children’s food consumption. J Dev Behav Pediatr.

[14] Bevelander KE, Anschütz DJ, Engels RCME (2012). Social norms in food intake among normal weight and overweight. Appetite.

[15] Reverdy C, Schlich P, Köster EP, Ginon E, Lange C (2010). Effect of sensory education on food preferences in children. Food Qual Prefer.

[16] Australian Government (2018). Qualification details diploma of early childhood education and care.

[17] Australian Government (2018). Qualification details certificate III of early childhood education and care.

[18] Department of Health and Ageing (2009). Get up and grow, healthy eating and physical activity for early childhood.

[19] Hallam Rena A., Fouts Hillary N., Bargreen Kaitlin N., Perkins Kelley (2014). Teacher–Child Interactions During Mealtimes: Observations of Toddlers in High Subsidy Child Care Settings. Early Childhood Education Journal.

[20] Alcock S (2007). Playing with rules around routines: children making mealtimes meaningful and enjoyable. Early Years.

[21] Mortlock A (2015). Toddlers’ use of peer rituals at mealtime: symbols of togetherness and otherness. Int J Early Years Educ.

[22] Brennan M (2007). A culture of tenderness: teachers’ socialisation practices in group care settings. Eur Early Child Educ Res J.

[23] Dotson HM, Vaquera E, Cunningham SA (2015). Sandwiches and subversion: teachers’ mealtime strategies and preschoolers’ agency. Childhood.

[24] Charmaz K (2014). Constructing grounded theory.

[25] Theobald M (2017). Children as research participants in educational research using video-stimulated accounts. Int J Educ Res.

[26] Siraj-Blatchford I. Conceptualising progression in the pedagogy of play and sustained shared thinking in early childhood education: a Vygotskian perspective. Educ Child Psychol. 2009;26(2):77–89.

[27] Edwards S (2017). Play-based learning and intentional teaching: forever different?. Australas J Early Childhood.

[28] Seuss (2010). Green eggs and ham, 50th anniversary edn.

[29] Holsten JE, Deatrick JA, Kumanyika S, Pinto-Martin J, Compher CW (2012). Children’s food choice process in the home environment. A qualitative descriptive study. Appetite.

[30] Hawkes Corinna, Smith Trenton G, Jewell Jo, Wardle Jane, Hammond Ross A, Friel Sharon, Thow Anne Marie, Kain Juliana (2015). Smart food policies for obesity prevention. The Lancet.

[31] Corsaro WA (2015). The sociology of childhood.

[32] Cromdal J, Brown K (2006). Socialization. Encyclopedia of language and linguistics.

[33] Oswell D (2013). The agency of children: from family to global human rights.

[34] Birch LL, Burns AC, Parker L, Institute of Medicine . Committee on Obesity Prevention Policies for Young C, Institute of M (2011). Early childhood obesity prevention policies.

[35] Scaglioni S, Arrizza C, Vecchi F, Tedeschi S (2011). Determinants of children's eating behavior. Am J Clin Nutr.

[36] Sobal J, Bisogni CA (2009). Constructing food choice decisions. Ann Behav Med.

[37] Sobal J, Nelson MK (2003). Commensal eating patterns: a community study. Appetite.

[38] Giacoman C (2016). The dimensions and role of commensality: a theoretical model drawn from the significance of communal eating among adults in Santiago, Chile. Appetite.

[39] Wiles R (2013). What are qualitative research ethics?.

